# Hydration for Clean Air Today^a^

**DOI:** 10.1142/S252973252101001X

**Published:** 2022-03-07

**Authors:** David A. Edwards, Bengt Norden, Lorie Karnath, Omar Yaghi, Chad J. Roy, Donald Johanson, Melanie Ott, John Brownstein, John Grove, Goran Tomson, Peter Friberg

**Affiliations:** 1 School of Engineering and Applied Sciences, Harvard University, Cambridge, MA;; 2 Chalmers University of Technology, Gothenburg, Sweden;; 3 Next Breath, Cambridge, MA;; 4 Stanford University, Palo Alto, CA;; 5 School of Medicine, Tulane University, New Orleans, LA;; 6 Institute of Human Origins, Arizona State University, Tempe, AZ;; 7 Gladstone Institute, University California San Francisco, San Francisco, CA;; 8 Children’s Hospital, Harvard Medical School, Boston, MA;; 9 World Health Organization, Geneva, Switzerland;; 10 Swedish Institute for Global Health Transformation (SIGHT), Stockholm, Sweden;; 11 Karolinska Institute, Stockholm, Sweden;; 12 Sahlgrenska Academy, Gothenburg University, Sweden;; 13 Gothenburg University, Gothenburg, Sweden.

Billions of years of human life are at risk of being lost by the breathing of dirty air before the COP26 zero emission target date of 2050. Most vulnerable are the world’s poorest, who are also at severe risk of dehydration^1^.

Threatened by various mega-trends^2^ including the droughts, heat waves, and air pollution accompanying global climate change—while living in circumstances where hand washing, use of clean masks, and access to indoor air filtration are unlikely on a wide scale—the poorest of the world face the possibility of catastrophic loss of life in the near term. The health challenge of poor access to clean air is compounded by a lack of clean water in almost one-third of the world and the continued burning of fossil fuels. In many parts of the world, climate change has altered weather patterns in ways where those relying on livestock and farming for their livelihood are no longer able to sustain their day-to-day activities^2^.

Even if nations find the resolve to keep the earth habitable against the assault of industrialization, foreseen with remarkable accuracy by Svelte Arrhenius in *The Philosophical Magazine* nearly 125 years ago, many millions will continue to die prematurely each year given these mega-trends. More than 90% of these deaths occur in low- and middle-income countries according to The Lancet Commission, with children and pregnant mothers at greatest risk^3^.

There is an urgent need for mass-scale translation of science to deliver clean air to the poorest of the world in water-scarce conditions. Success will require public–private investment in (1) the testing, health-benefit validation, scaling, and distribution of practical technologies that provide inexpensive clean air and freshwater access; (2) the remote detection and surveillance of respiratory and dehydration symptoms of at-risk populations; and (3) the education of children in at-risk regions to enhance adoption and compliance of new technologies and practices—and ultimately to save lives.

## SCIENCE TRANSLATION

Science continues to improve the design and function of nonpharmaceutical technologies such as face masks, air filtration, and germicidal UV for cleaning of air in commercial markets, sparked by the COVID-19 pandemic^4,5^. Adoption and integration, of these and other simple technologies, each a beneficial response to the air quality challenges of climate change, has accelerated over the last two years among high- and middle-income populations through a combination of public discourse and mandates, commercial markets, and charitable giving.

Similar action and coordination is needed in low-income settings where simple and inexpensive solutions will need to adapt to harsher circumstances, and where the challenges of dehydration and breathing polluted air are indeed interrelated.

When dehydrated, people face greater health risks on the breathing of dirty air. Dehydration dries out the larynx, trachea, and main bronchi, which clean and hydrate inhaled air, reducing cilia beat frequency, damaging epithelial cells^6^, and amplifying respiratory droplets that carry inhaled contaminants—allergens, pathogens, and carcinogens—deeper into the lungs^7^. Loss of water in the upper airways, simultaneously aggravated by whole-body dehydration and the dryness of inhaled air, increases risks of chronic and acute airborne respiratory diseases^6,8^. Rapid global increase of respiratory disease-associated morbidity and disability-adjusted life years (DALYs) (i.e., the sum of years of potential life lost due to premature mortality and the years of productive life lost due to disability) reflects these circumstances^9^, notably in at-risk communities characterized by high levels of airborne particulates, ozone, and oxides of nitrogen and sulfur. Physiologically compromised lungs resulting from continuous environmental insult will naturally be more susceptible to airborne respiratory disease, thereby perpetuating endemic and pandemic disease.

New materials that deliver water to families and communities with little access^10^, simple methods for elevating humidity of inhaled air as in the wearing of facial masks^4^, and accessible new hygiene rites to keep water in the airways of those at risk of dehydration and the breathing of dirty air^11^ are among the new science insights and technologies emerging out of the COVID-19 pandemic with the practical potential to help save lives of those most at risk today. Simple, practical, and effective technology for whole-body and airway hydration should be evaluated in large-scale randomized controlled studies and implementation field tests, and where promising should be rapidly deployed at scale.

Distribution of these new technologies and practices to some of the most remote communities, assessment of compliance, maintenance of function, and assurance of adoption are challenges that have almost no precedent in the translation of new technology in high-income settings. As the effectiveness of practical hydration to improve respiratory health is established, there will be a need to coordinate adoption that is at least as ambitious as recent pandemic efforts to advance effective hygienic behavior and promote vaccine distribution in the most advanced countries of the world.

## DETECTION AND SURVEILLANCE

Digital technologies^12^ that monitor respiratory health, as in the registering of coughs and laryngeal dehydration^13^, will increasingly permit remote healthcare providers to understand the prevalence and nature of respiratory disease of those who rarely, if ever, show up in a clinical setting. Responding to health needs in the field with digital point-of-care, low-cost diagnostic technologies, many of which have rapidly advanced during the pandemic, will increasingly permit treatment outside of the clinic, as in the amplification-free detection of SARS-CoV-2 with CRISPR-Cas13a and mobile phone microscopy^14^.

Digital surveillance of air quality, of disease outbreaks, and of societal response to public health measures and communiqués are all advancing rapidly and will be helpful in further managing the respiratory health of those least cared for by the healthcare system^15^.

## EDUCATING CHILDREN

Of the 1.6 billion school-age children around the world, an estimated 1 billion are living in multi-dimensional poverty^16^ with respiratory disease their principal threat of illness and death^1^. They are also most likely to respond to education— and to access health and hygiene interventions in school-based settings.

Delivering clean air and fresh water to those living today in parched dirty air settings will require an extraordinary effort on behalf of public and private sectors; and a fundamental change in the behavior of those most at risk. We generally know when we are thirsty. We rarely know when we are in need of clean hydrated air.

Children should be taught what it means to breathe clean air, and why it matters. Children and their teachers should be made aware that the natural clearance function of our lungs evolved in humid, clean, and salty air environments, and that in the dry and dirty air environments of today, natural clearance function can be restored by the wearing of masks or face coverings^4^, and the breathing into the upper airways of salt water^11^. Learning about the air they breathe, children can discover the interconnectedness of clean air to the water they drink, the food they eat, and the need to maintain clean air and clean water in the regeneration of our ecosystems.

Education providers and charities should aim to move simple scientific advances in the hydration and oxygenation of the poorest into the field with educational programs that make the current generation of youth the smartest where it comes to the air we breathe.

## THE URGENCY OF ACTION

Twenty-eight years ago, the Harvard Six City Study^17^ determined that an increase of fine particulate matter (PM 2.5) from 10 to 30 μg/m^3^ reduced human longevity by approximately three years. By contrast, fine particulate levels in New Delhi over recent days averaged 340 μg/m^3^. New York City averaged 93 μg/m^3^.

We call for urgent action to improve the inhaled air quality of human populations most at risk today by (1) science translation of low-cost interventions that effectively hydrate the airways topically and systemically; (2) development and implementation of reliable detection and surveillance to ensure adoption of new hydration practices outside the healthcare system; and (3) the design and implementation of educational environments suited to the most rugged conditions to effectively manage technology translation and behavior change through childhood education.

A moonshot to save billions of life years on the planet through effective hydration does not reduce the imperative to reduce carbon emissions toward the 2050 goals^18^. Success at keeping the earth habitable for generations to come will require changing behavior across populations, mobilizing and coordinating public and private sectors, and coordinated government action. Collectively, we need to continue our transition to natural renewable energy sources, such as solar energy harvested at sunny latitudes, and hydrogen by the electrolysis of water. We need global collaborations, such as solar energy export; and we need to galvanize public interest in the face of the many sacrifices that will be needed to achieve zero carbon dioxide missions.

The 2021 report of the Lancet Countdown on health and climate change points to “an unprecedented opportunity” to ensure a healthy future for all^19^. Meanwhile, to realize our opportunity, we need clean breaths today. With the COVID-19 pandemic catalyzing behavior change, we should seize this moment to improve human and planetary health and well-being^20^ with the aim of building a more sustainable future.
